# F.E.M. Stress-Investigation of Scolios Apex

**DOI:** 10.2174/1874120701812010090

**Published:** 2018-10-25

**Authors:** A. Daghighi, H. Tropp, N. Dahlström, A. Klarbring

**Affiliations:** 1 Department of Clinical and Experimental Medicine, Linköping University, 581 83 Linköping, Sweden; 2 Department of Clinical and Experimental Medicine & Division of Surgery Orthopedics and Oncology, Linköping University, 581 83 Linköping, Sweden; 3 Center for Medical Image Science and Visualization, Linköping University, 581 83 Linköping, Sweden; 4 Department of Management and Engineering, Division of Solid Mechanics, Linköping University, 581 83 Linköping, Sweden

## F.E.M. Stress-Investigation of Scolios Apex

The Open Biomedical Engineering Journal, **2018**, 12: 51-71

The author has removed the provided Telephone Number.

